# Breathing Pure Oxygen

**Published:** 1888-01

**Authors:** 


### Breathing Pure Oxygen.

According to the *Popular Science News*, Dr. B. W. Richardson, of England, in making some investigations upon the physiological effects of breathing pure oxygen by various animals, has discovered that, by simply passing the gas a few times through the lungs, it becomes "devitalized," or incapable of supporting life, although its chemical composition remains the same, and all carbonic dioxides and other impurities are removed. He also found that by passing electric sparks through the gas it became "revitalized," and regained its usual stimulating effect upon the animal economy. The devitalized oxygen would still support life in cold-blooded animals, and combustible bodies would burn in it as brilliantly as ever. Dr. Richardson considers that while the gas is in contact with the tissues or blood of a warm blooded animal some quality essential to its life supporting power is lost.

